# A study of patient satisfaction after cataract surgery with implantation of different types of intraocular lenses

**DOI:** 10.1186/1756-0500-5-592

**Published:** 2012-10-29

**Authors:** Ching-Kuo Wei, Shun-Mu Wang, Jen-Chieh Lin

**Affiliations:** 1 Department of Health Care Administration, Oriental Institute of Technology, Taipei, Taiwan; 2 Department of Ophthalmology, Taipei City Hospital, Heping Fuyoy Branch, Taipei, Taiwan; 3 Graduate Institute of Epidemiology & Preventive Medicine, College of Public Health, National Taiwan University, Taipei, Taiwan

## Abstract

**Background:**

The implementation of capitated payment has driven medical institutions through developing balance billing for medical services. By exploring the patients’ decision-making factors on different self-pay items, a reference for the pricing and sales strategy for the related products can be formed. The major purposes of this study were to analyze the determinants of preoperative selection and postoperative satisfaction with implantation of different types of intraocular lenses in cataract surgery.

**Methods:**

This cross-sectional study consisted of 127 patients that were 50 years of age and older, and who had phacoemulsification with intraocular lens implantation in both eyes. Data were collected by using a structured questionnaire. The following parameters were measured: access to medical care, attitude towards receiving medical products at one’s own expense, overall patient satisfaction and postoperative visual clarity.

**Results:**

The results showed that the patient’s gender, educational level and economic status influenced the type of intraocular lens chosen. Patients in the insurance group cared about access to medical care, and patients in the balance billing group cared about product differentiation. ANOVA results showed no statistically significant differences in the overall satisfaction of the patients among the groups with different types of intraocular lenses. Patients that received cataract surgery with implantation of multifocal intraocular lenses had better vision when trying to view smaller objects and when looking at objects under strong light.

**Conclusions:**

Manufacturers should increase the number of differences between their products, and health care providers can then recommend the appropriate intraocular lens in accordance with the needs or demands of their patients, and also by keeping in mind the financial constraints of their patients.

## Background

The National Health Insurance (NHI) program, a form of compulsory universal health insurance, was implemented in Taiwan on March 1, 1995. It has reduced the public’s financial burden and enhanced accessibility to healthcare. However, the implementation of capitated payment has limited the portion of the income from the NHI. As such payments are likely to be further reviewed and tightened, medical institutions are actively developing medical services that are not paid by the NHI placing more of a focus on self-paying patients [1].

According to the Rational Decision-making Model by Stratmann [2], there are 5 different decision-making factors for healthcare users: 1) economic factor; 2) time factor; 3) convenience factor; 4) social psychological factor; and 5) quality of medical services factor. There have been several studies related to the factors for the selection of self-pay medical services, and the popularity of the hospitals and physicians has been found to have a significant influence. In addition, medical service procedures, convenience, doctor’s recommendations, medical skills, costs, and the medical environment will affect the public’s decision when considering whether or not to choose self-pay medical services. Individual traits, type of disease, age, education level, and place of residence will also affect this type of selection [3-6].

Other than general self-pay medical services, the Bureau of National Health Insurance (BNHI) has opened access to the option of “balance billing” for medical services; that is, the NHI will provide partial payment towards a special medical service [7]. Among such services eligible for balance billing offered by the NHI, intraocular lenses (IOL) with special functions have been included since October 1, 2007. As numerous types of medical services and devices are offered by the balance billing system and the room for self-pay by patients increases, more patients are willing to use this option.

Cataracts lead to impairment of activities of daily living and may even cause blindness. Surgical removal of the cataract is thus the only effective treatment, with phacoemulsification and intraocular lens implantation being the most common technique in high-income countries [8,9]. The expected result is not only enhanced visual acuity, but also, in most cases, an improvement in the patient’s quality of life [10].

The “general IOL” covered in the NHI system is composed of PMMA, silicone or acrylic; basically a spherical IOL. Patients can only see objects at a fixed distance with this type of device, and have to wear reading glasses in order to see objects at various distances. Further, another drawback of spherical IOLs is poor night vision that may cause inconvenience for those who are active at night [11,12]. Special functional IOLs can enhance visual acuity, reduce blurring at night or under insufficient lighting, correct astigmatism, increase visible distance, and address the drawback of the loss in adjustment while providing the benefits of blue-light filtration, lighting softening and vision enhancement.

There are many types of special functional IOLs available in Taiwan, which can be categorized as monofocal, multifocal, yellow-tinted, astigmatism-correction, as well as others types with additional functions. The NHI pays USD$92, the pricing for a “general IOL”, and those who wish to use a “special functional IOL” have to cover the difference [7].

The balance of payment for each functional IOL varies greatly depending on the type, pricing and functions, and ranges from USD$388-2265 [7]. Therefore, by exploring the patients’ decision-making factors on different self-pay items, a reference for the pricing and sales strategy for the related products can be formed. The major purposes of this study were to explore the decision-making factors patients consider with regards to various types of IOL, to explore the relationship between patients with different characteristics and the type of IOL chosen, and to explore the variation in postoperative satisfaction of the patients receiving different types of IOL.

## Methods

### Study population

Patients presenting for follow-up examinations at two private clinics and one regional hospital in Taipei between October 1, 2009 and December 31, 2009 were eligible for the study if they were over 50 years of age. All patients had uneventful phacoemulsification of both eyes with a temporal clear corneal incision or superior scleral tunnel incision, a curvilinear capsulorhexis, and in-the-bag IOL placement. All participants were interviewed one-to-one by a single interviewer to complete the survey. A structured questionnaire was developed to investigate the factors initially considered during the selection of self-pay products, postoperative satisfaction and visual clarity. The study was approved by an Investigational Review Board of Taipei City Hospital and was in accordance with the tenants of the Declaration of Helsinki. We also obtained informed consent from all patients before study initiation.

### Contents of the questionnaire

*IOL implantation*: for the purpose of this research, questions such as the acceptance of self-pay medical services and self-pay IOL, the IOL selected [NHI covered, aspherical monofocal IOL, yellow-tinted multifocal IOL(Acrysof ReSTOR SN60D3), multifocal IOL(AMO Tecnis ZM900), yellow-tinted monofocal IOL], amount of balance billing, postoperative recovery time, source of information, selection criteria (multiple choice), and post-operative concerns (multiple choice) were examined.

### Selection factors

A total of 13 questions were developed to investigate the factors for consideration. The questionnaire was scored using the Likert 5-point scale awarding 5 to 1 points for answers with “mostly agree,” “agree,” “average,” “disagree,” or “mostly disagree.”

### Satisfaction evaluation

A total of 10 questions were developed to investigate the satisfaction of the treatment process. The questionnaire was scored using the Likert 5-point scale awarding 5 to 1 points for answers with “very satisfied,” “satisfied,” “average,” “unsatisfied,” or “very unsatisfied.”

### Postoperative visual clarity evaluation

A total of 10 questions were developed to evaluate current visual clarity. The questionnaire was scored using the Likert 5-point scale awarding 5 to 1 points for answers with “very clear,” “clear,” “average,” “unclear,” or “very unclear.”

### Demographic variables

Included gender, age, education level, marital status, household monthly income, and occupation.

### Statistical analysis

Data analysis was performed using SPSS 12.0 for Windows (SPSS Inc., Chicago, IL, USA). Descriptive statistics were reported as number (%), mean [standard deviation (SD)] and proportion (%). Independent t-testing, one-way ANOVA and multivariate logistic regression were performed to evaluate the relationship in the decision-making, satisfaction, and postoperative visual clarity to the selection of various types of IOL. Cross tabulation with the chi-square test was performed to determine whether there were significant differences in the selection of an IOL by different demographic variables. Internal consistency of reliability of the applied questionnaire was assessed using Cronbach’s testing.

## Results

One hundred and twenty-seven patients were included in the study, including 82 (64.6%) females. The demographics and clinical data for the participants are presented in Table 1. 44.9% of the participants were aged above 70 years and 37.8% aged 61 to 70 years. With respect to IOL selection, 49 (38.6%) patients chose an IOL covered by the NHI, and the special-functional IOLs most commonly chosen were aspherical monofocal IOLs (24.4%), followed by yellow-tinted multifocal IOLs (18.9%), multifocal IOLs (11.0%), and yellow-tinted monofocal IOLs (7.1%). The mean balance billing was USD$1522. 30.7% of the sources of information for the choice of IOL implantation came from family and friends, and 64.6% from physicians. Cronbach’s α was 0.87 or higher for all subscales of the questionnaire, and the expert validity was 4.03.

**Table 1:** Demographic and clinical characteristics of the study subjects

**Variable**	**Number**	**%**	**Variable**	**Number**	**%**
Gender			Household monthly income, US$		
Male	45	35.4	<1300	63	49.6
Female	82	64.6	1300-2600	32	25.2
Age (years)			>2600	32	25.2
≤60	22	17.3	IOL selected		
61-70	48	37.8	NHI covered	49	38.6
>70	57	44.9	Aspherical monofocal IOL	31	24.4
Marital status			Yellow-tinted multifocal IOL	9	7.1
Married	107	84.2	Multifocal IOL	14	11.0
Non-married	20	15.8	Yellow-tinted monofocal IOL	24	18.9
Educational level			Information source for IOL implantation		
<Primary	47	37.0	Family and friends	39	30.7
High school	54	42.5	Physician	82	64.6
>College	26	20.5	Mass media	6	4.7
Working status					
Working	29	22.8			
Not working	98	77.2			

### The influence of demographic variables on the selection of medical service

The chi-square test was used in this study to investigate whether the demographic variables had an influence on the selection of the medical services. The sub-categories of the self-pay IOLs were combined into monofocal and multifocal groups to help with the implementation of the test. The results showed that level of education and household monthly income affected the selection of medical service (p < 0.01). Those with a college education or above tended to choose a multifocal IOL, while those with elementary education or below tended to choose the IOL covered by the NHI. Those with a higher household monthly income tended to select a multifocal IOL, while those with a lower income tended to use the IOL covered by the NHI.

### The factors influencing the selection of medical service

We categorized the subjects into NHI coverage and balance billing in order to explore the variation in the factors concerning medical services (Table 2). The results showed a statistically significant difference in two items: accessibility to medical care and the difference between self-pay medical products. Those who selected the NHI coverage viewed accessibility as an important factor, while those who selected balance billing were more concerned with the differences among the self-pay products.

**Table 2:** The differences in choosing medical services between NHI coverage and balance billing groups

		**Number**	**Mean**	**SD**	**T-test**	**P-value**
Access to medical care	NHI coverage	49	4.06	0.94	2.387	0.019
	Balance billing	78	3.65	0.92		
Product differentiation	NHI coverage	49	3.79	0.95	−2.665	0.009
	Balance billing	78	4.16	0.59		

Notes: The mean was calculated from a Likert Scale from 5 (very important) to 1 (not very important).

### The influence of various types of IOLs on postoperative satisfaction and visual clarity

Table 3 shows the differences in postoperative satisfaction of the selection of different types of IOLs. The only statistically significant difference was in regards to the attitudes of medical staff. A higher satisfaction was observed in those who chose yellow-tinted multifocal and NHI-covered IOLs; however, no statistically significant difference was observed in the post hoc test. Generally speaking, those who selected the NHI-covered IOL gave a higher score for the professionalism of the physicians, attitude of the medical staff, interpretation ability of the physicians, overall satisfaction with the surgery, and postoperative comfort, than those who chose balance billing. In terms of postoperative vision and product quality, there was a higher satisfaction score in those who chose multifocal and yellow-tinted multifocal IOLs, but this did not reach statistical significance.

**Table 3:** The differences in the postoperative satisfaction on the selection of different types of intraocular lenses

		**Number**	**Mean**	**SD**	**F test**	**P-value**
Professionalism of the physicians	monofocal-aspheric	31	4.74	0.51	1.076	0.372
	monofocal-yellow	9	4.44	0.53		
	multifocal	14	4.57	0.65		
	multifocal-yellow	24	4.67	0.48		
	NHI covered	49	4.78	0.51		
Attitudes of the medical staff	monofocal-aspheric	31	4.45	0.62	2.732	0.032
	monofocal-yellow	9	4.22	0.44		
	multifocal	14	4.50	0.52		
	multifocal-yellow	24	4.71	0.46		
	NHI covered	49	4.71	0.50		
Interpretation ability of the physicians	monofocal-aspheric	31	4.52	0.63	1.286	0.279
	monofocal-yellow	9	4.67	0.50		
	multifocal	14	4.50	0.65		
	multifocal-yellow	24	4.67	0.48		
	NHI covered	49	4.76	0.43		
Overall medical service process	monofocal-aspheric	31	4.35	0.61	1.329	0.263
	monofocal-yellow	9	4.44	0.53		
	multifocal	14	4.50	0.65		
	multifocal-yellow	24	4.67	0.48		
	NHI covered	48	4.60	0.57		
Overall satisfaction with the surgery	monofocal-aspheric	31	4.39	0.72	1.161	0.331
	monofocal-yellow	9	4.22	0.44		
	multifocal	14	4.57	0.65		
	multifocal-yellow	24	4.58	0.58		
	NHI covered	49	4.61	0.64		
Postoperative vision	monofocal-aspheric	31	4.16	0.86	1.730	0.148
	monofocal-yellow	9	3.78	0.67		
	multifocal	14	4.50	0.65		
	multifocal-yellow	24	4.17	0.76		
	NHI covered	49	4.39	0.79		
Postoperative comfort	monofocal-aspheric	31	4.19	0.83	0.827	0.511
	monofocal-yellow	9	4.00	0.50		
	multifocal	14	4.36	0.74		
	multifocal-yellow	24	4.21	0.72		
	NHI covered	49	4.41	0.79		
Medical facilities	monofocal-aspheric	31	4.32	0.65	1.016	0.402
	monofocal-yellow	9	4.33	0.50		
	multifocal	14	4.57	0.65		
	multifocal-yellow	24	4.58	0.58		
	NHI covered	49	4.35	0.63		
Product quality	monofocal-aspheric	31	4.29	0.69	1.194	0.317
	monofocal-yellow	9	4.33	0.50		
	multifocal	14	4.57	0.65		
	multifocal-yellow	24	4.58	0.58		
	NHI covered	49	4.31	0.68		

Notes: The mean was calculated from a Likert Scale from 5 (very satisfied) to 1 (not very satisfied).

To understand the influence of different types of IOLs on postoperative visual clarity, this study found no statistically significant difference in subject analysis on the selection of different types of IOLs. With regards to the postoperative visual clarity of the subjects who chose IOLs that required balance billing, there were statistically significant differences in two items: looking at small objects and looking at objects under strong light (Table 4). A higher subjective visual clarity was found in multifocal IOLs, but there was no significant difference in the post hoc test. With regards to looking at objects under strong light, post hoc tests showed that the subjective clarity of those who selected yellow-tinted multifocal IOLs was higher than those who chose monofocal IOLs.

**Table 4:** The differences in the postoperative visual clarity among different types of intraocular lenses

		**Number**	**Mean**	**SD**	**F test**	**P-value**
Reading a newspaper	monofocal-aspheric	30	3.83	0.91	0.593	0.669
	monofocal-yellow	9	3.56	0.88		
	multifocal	14	4.14	0.86		
	multifocal-yellow	24	3.96	0.86		
	NHI covered	49	3.90	1.03		
Viewing a computer screen	monofocal-aspheric	29	3.86	0.92	0.159	0.958
	monofocal-yellow	8	4.13	0.64		
	multifocal	14	4.00	0.88		
	multifocal-yellow	24	3.92	0.93		
	NHI covered	49	3.93	0.90		
Watching television	monofocal-aspheric	30	4.10	0.76	0.542	0.705
	monofocal-yellow	9	3.89	0.60		
	multifocal	14	4.14	0.77		
	multifocal-yellow	24	3.96	0.95		
	NHI covered	49	4.20	0.82		
Night driving	monofocal-aspheric	23	3.91	0.79	0.670	0.614
	monofocal-yellow	8	3.38	0.52		
	multifocal	14	3.64	0.63		
	multifocal-yellow	24	3.71	1.08		
	NHI covered	49	3.82	0.94		
Distance-vision activities	monofocal-aspheric	30	3.90	0.80	0.223	0.925
	monofocal-yellow	9	3.67	0.71		
	multifocal	14	4.00	0.96		
	multifocal-yellow	24	3.79	0.98		
	NHI covered	49	3.86	1.00		
Looking at small objects	monofocal-aspheric	30	3.30	0.99	1.926	0.110
	monofocal-yellow	9	3.00	0.50		
	multifocal	14	3.93	0.92		
	multifocal-yellow	24	3.75	0.90		
	NHI covered	49	3.56	1.11		
Looking at objects under strong light	monofocal-aspheric	30	3.23	0.77	2.080	0.088
	monofocal-yellow	9	3.33	0.71		
	multifocal	14	3.79	0.89		
	multifocal-yellow	24	3.88	0.74		
	NHI covered	49	3.57	1.06		
Color discrimination	monofocal-aspheric	29	4.21	0.73	0.682	0.606
	monofocal-yellow	9	3.78	0.67		
	multifocal	14	4.14	0.53		
	multifocal-yellow	24	4.08	0.72		
	NHI covered	49	4.20	0.87		
Looking at moving objects	monofocal-aspheric	30	4.00	0.91	0.385	0.819
	monofocal-yellow	9	3.78	0.67		
	multifocal	14	4.14	0.66		
	multifocal-yellow	24	3.92	0.72		
	NHI covered	49	4.06	0.91		
Dry eye sensation	monofocal-aspheric	30	3.23	1.04	1.117	0.352
	monofocal-yellow	9	3.22	0.83		
	multifocal	14	3.71	0.83		
	multifocal-yellow	24	3.75	0.99		
	NHI covered	49	3.51	1.17		

Notes: The mean was calculated from a Likert Scale from 5 (very clear) to 1 (not very clear).

Multiple logistic regression analysis identified five factors associated with the selection of non-NHI covered IOLs: 1) male gender; 2) education level of college and above; 3) household monthly income > US$2600; 4) quality of postoperative vision; and 5) postoperative comfort (Table 5).

**Table 5:** Multiple logistic regression analysis of factors associated with preoperative selection of non-NHI covered intraocular lenses

**Variable**		**OR (95% CI)**	***P*-value**
Gender	Male	4.035(1.227,13.270)	0.022*
	Female	-	-
Age (years)	≤60	1.396(0.293,6.652)	0.675
	61-70	2.063(0.419,10.151)	0.373
	>70	-	-
Marital status	Married	0.828(0.212,3.232)	0.786
	Non-married	-	-
Educational level	<Primary	-	-
	High school	2.532(0.768,8.353)	0.127
	>College	8.204(1.398,48.130)	0.020*
Household monthly income, US$			
	<1300	-	-
	1300-2600	0.866(0.266,2.816)	0.811
	>2600	6.970(1.366,35.551)	0.020*
Working status	Working	0.299(0.075,1.191)	0.087
	Not Working	-	-
Postoperative vision		3.873(1.144,13.112)	0.030*
Postoperative comfort		4.548(1.349,15.332)	0.015*

“-“ Indicates the reference category.

* Significantly correlated with outcome, P<0.05.

## Discussion

### Exploring the factors used by the patients in the selection of different types of IOLs

Most factors used by the patients in the selection of the different types of IOL did not show significant differences. However, we did find that those who selected balance billing placed less emphasis on accessibility to medical care, and a greater emphasis on the differences among the self-pay products. It is reasonable to assume that those who chose balance billing expected better results from an IOL than those provided by the IOL covered by the NHI, and were therefore more accepting of self-pay products.

### Exploring the relationship between patients with different profiles and the choice they made in the selection of different types of IOL

With regards to demographic variables, patients with a higher education level and higher household monthly income tended to choose an IOL that required balance billing. The cost of the IOL covered by the NHI is only USD$92, but IOLs requiring balance billing cost far more, typically over USD$2000 for a multifunctional IOL. The self-pay expense is extremely high if the implantation of an IOL into both eyes is taken into consideration, and those without an established economic base would not be able to afford such a burden. The monthly household income was usually higher for those who had a better education, and also for those more accepting of new medical technology, and therefore they were more likely to use the higher-priced multifunctional IOLs.

### Exploring the differences in postoperative satisfaction for patients receiving different types of IOL implantation

There were not many differences in terms of satisfaction with medical care, however a statistically significant difference was noted for the attitude of the medical staff. The satisfaction ranked highest for those who chose NHI-covered and yellow-tinted IOLs. It is worth noting that the satisfaction of those who chose the NHI-covered IOL was not lower than those who selected balance billing. The reason may be the lower expectations of medical services for the NHI, and thus, the difference between the actual and expected medical services received was relatively minor, leading to better satisfaction. The self-pay patients had to endure a higher medical cost, and therefore, had higher expectations with regards to the medical services they were to receive; this may have lead to a lower degree of satisfaction when the actual service received was compared to what was expected. In terms of postoperative visual clarity, no difference was found between NHI-covered and balance billing IOLs. The subjective clarity of patients who selected multifocal and yellow-tinted multifocal IOLs was better in terms of looking at small objects and looking at objects under strong light.

For manufacturers, the results of this study show that the most important factor for patients when selecting self-pay products was product differentiation. However, there were no statistically significant differences in most other factors in a further comparison of the postoperative differences of the various types of IOLs. Those who selected the NHI-covered IOL even had a higher level of satisfaction than those who chose the multifunctional IOLs. The reason for this may be the difference in product expectation between the two; nevertheless, the low differentiation between the products is clear. Therefore, manufacturers should consider focusing on product development to enhance differentiation, making this more appealing to consumers and more apparent. The results of the survey found that patients were most concerned about postoperative visual stability, visual recovery, and recurrence of the lens opacity. Therefore, focus should be placed on these three points to enhance product appeal. Patients receive information regarding IOL implantation mostly from their doctors; therefore, product education for the physicians should be comprehensive. In product differentiation, multifocal IOLs have the ability to adjust focus, so the clarity is better than that of a monofocal IOLs when looking at objects, and especially small objects. Yellow-tinted IOLs have a shading capability, and therefore can offer clarity when viewing objects under strong lighting [13,14]. When inquiring about IOLs with different functions, physicians should be able to provide appropriate recommendations by understanding the need to look at small objects or looking at objects under strong lighting. In addition, since the pricing of multifunctional IOLs is higher, the recommendations should also consider the patient’s economic status.

For health authorities, providing products that require balance billing is an important strategy for the future. However, this study discovered that the NHI-covered IOL is already sufficient to meet the needs of most patients. In addition, no statistically significant difference was found with regards to medical care satisfaction and postoperative visual clarity between the NHI-covered and self-pay IOLs. This shows that the NHI-covered IOL still provides a certain degree of quality. From this perspective, the medical services provided by the NHI can satisfy the needs of most patients. The original intention of offering balance billing by the BNHI was to reduce the economic burden on patients when selecting self-pay products that are usually more expensive. However, the price difference between the multifunctional and NHI-covered IOLs is high, and the general public may still be unable to take on this burden directly. The BNHI has begun evaluating the inclusion of many other medical devices into balance billing. However, careful consideration should be given to the actual demand and whether the benefits of balance billing can be enjoyed by the majority of the general public.

We suggest that future research can be conducted to obtain more samples in order to understand the characteristics of people seeking medical care from different sectors. In addition, studies on other products requiring balance billing should be conducted to compare the differences with the results from this study. Focusing on the differences in expectations between the NHI-covered and self-pay IOLs, future studies could use the SERVQUAL scale to adjust the variation of each patient’s expectations, and this should be able to establish the real level of satisfaction felt by the patients selecting different medical services.

## Conclusions

Manufacturers should increase the number of differences between their products, and health care providers can then recommend the appropriate intraocular lens in accordance with the needs or demands of their patients, and also by keeping in mind the financial constraints of their patients.

## Competing interests

The authors declare that they have no competing interests.

## Authors' contributions

JL was involved in this study’s conception and design, analysis and interpretation of data, and drafting the manuscript. SW was involved in this study’s conception and design, acquisition of data, analysis and interpretation of data, and revising the manuscript. CW was involved in this study’s conception and design, analysis and interpretation of data, supervising the research team and revising the manuscript. All authors read and approved the final manuscript.
